# Follicular dendritic cell sarcoma of the stomach in a young male: A rare case report and literature review

**DOI:** 10.1097/MD.0000000000043004

**Published:** 2025-07-04

**Authors:** Xiujuan Sun, Chengyu Hu, Weihua Gong

**Affiliations:** a Department of Radiology, Hospital of Zhejiang University School of Medicine, Hangzhou, China; b Department of Surgery, Hospital of Zhejiang University School of Medicine, Hangzhou, China.

**Keywords:** case report, imaging, pathology, sarcoma, stomach

## Abstract

**Rationale:**

Follicular dendritic cell sarcoma (FDCS) is an exceptionally rare mesenchymal tumor, with gastric involvement being scarcely reported (only 7 prior cases). This case highlights the diagnostic challenges and underscores the importance of multimodal imaging in identifying this malignancy, particularly in extra-nodal sites like the stomach.

**Patient concerns:**

A 25-year-old male presented with acute abdominal pain and a palpable mid-abdominal mass. He denied weight loss, fever, or gastrointestinal symptoms. Laboratory tests revealed elevated squamous cell carcinoma-related antigen (3.6 ng/mL).

**Diagnoses:**

Contrast-enhanced abdominal computed tomography (CT) revealed a 68 × 47 × 91 mm soft tissue mass in the gastric antrum, vascularized by the left gastric artery. Positron emission tomography/CT demonstrated intense FDG uptake (SUVmax 25.48). Magnetic resonance images showed a T1-isointense, T2-hypointense mass with diffusion restriction (diffusion weighted imaging hyperintensity, low ADC) and moderate arterial enhancement. Endoscopy identified a submucosal gastric lesion. Histopathology confirmed FDCS via spindle/epithelioid cells, neutrophils, and immunohistochemistry (CD23/CD35/CD31+, S100/CK−).

**Interventions:**

The patient underwent distal gastrectomy with Roux-en-Y gastrojejunostomy. Intraoperatively, the tumor was resected with clear margins, preserving gastric function. No lymph node metastasis was observed.

**Outcomes:**

Postoperative recovery was uneventful. Pathology confirmed a 9 cm FDCS without necrosis or calcifications. Immunohistochemistry validated the diagnosis. No adjuvant therapy was administered, and the patient remained recurrence-free during follow-up.

**Lessons:**

Gastric FDCS is easily misdiagnosed as gastrointestinal stromal tumor (GIST) or Castleman disease due to overlapping imaging features. Multimodal imaging (CT, MRI, positron emission tomography/CT) combined with histopathology is critical for accurate diagnosis. Radical surgery remains the cornerstone of treatment, emphasizing the need for wide resection to minimize recurrence. This case provides the first comprehensive imaging characterization of gastric FDCS, enhancing awareness and diagnostic precision for this rare entity.

## 1. Introduction

Follicular dendritic cell sarcoma (FDCS) originates from proliferating dendritic cells. It is an uncommon malignant tumor and reported by Monda et al in 1986 firstly.^[1]^ While FDCS primarily occurs in lymph nodes, cases in extranodal sites are less common. FDCS in extranodal locations is often misdiagnosed due to its rarity and lack of consideration in the differential diagnosis. Primary gastric FDCS cases are exceptionally rare, with only a few cases reported. In this case, we present a patient of FDCS originating in the stomach, which is the eighth reported case of gastric FDCS in literature.^[2–8]^ The aim of our study is to provide a literature review of this rare disease, focusing on clinical characteristics and imaging features to improve diagnostic accuracy. By sharing our findings, we hope to increase awareness and understanding of FDCS, particularly its occurrence in the stomach.

## 2. Case presentation

A 25-year-old man presented with abdominal pain for 10 hours, visited to the emergency department. He denied fever, nausea, vomiting and weight loss, with no remarkable past medical history and history of family disease. Laboratory test results showed squamous cell carcinoma-related antigen 3.6 ng/mL (normal range: < 1.5 ng/mL).

**Figure 1.**
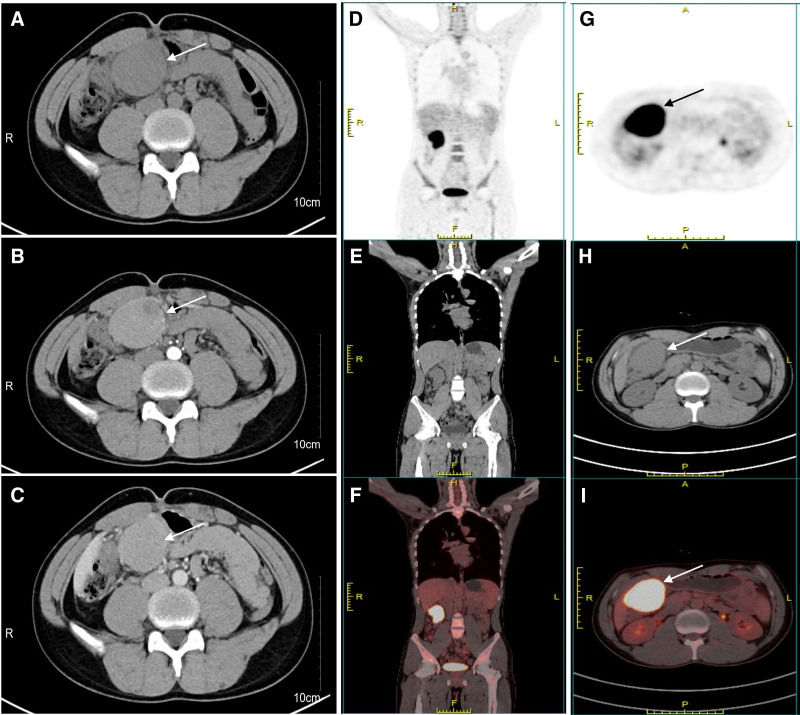
Computed tomography and 18F-FDG PET/CT image of gastric FDCS. (A) Unenhanced axial CT showed well-defined, homogeneously hypoattenuating mass (arrow) with approximately 68 mm × 47 mm × 91 mm in the right mid-abdomen. (B and C) Contrast-enhanced axial CT showed the tumor being heterogenous strengthened, enhanced scan continues to be strengthened. (D–I) focal hypermetabolism of the lesion corresponding to the mass in CT. CT = computed tomography, FDCS = follicular dendritic cell sarcoma, PET = positron emission tomography.

**Figure 2.**
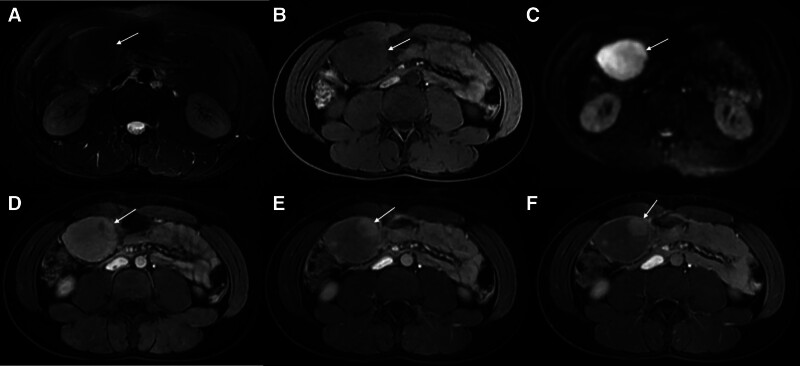
Magnetic resonance images image of gastric FDCS. (A) Axial T2-weighted MRI showed well-defined, hypointense mass (white arrow) in right mid-abdomen. (B) The mass was isointense on axial T1-weighted MRI. (C) On axial DWI, the mass was obviously hyperintense. (D–F) Axial T1-weighted contrast-enhanced MR images in arterial (D) portal (E) and delayed (F) phases showed uneven strengthening. DWI = diffusion weighted imaging, FDCS = follicular dendritic cell sarcoma, MRI = magnetic resonance images.

**Figure 3.**
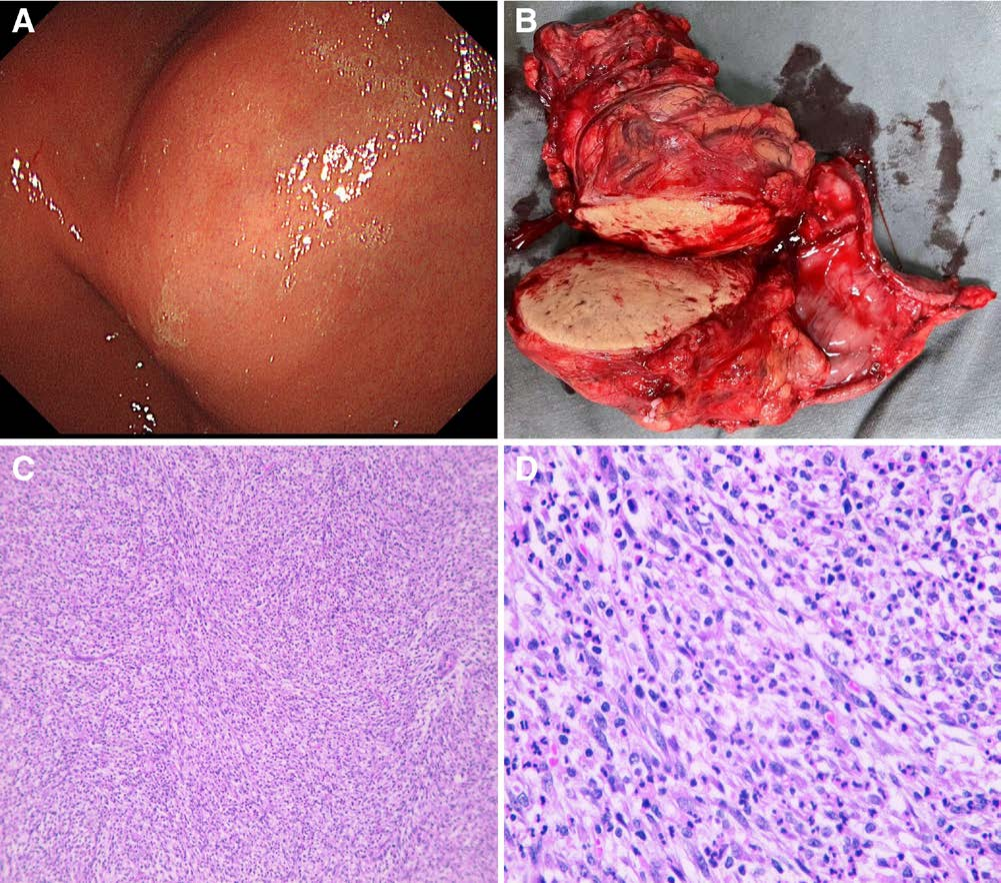
(A) Upper gastrointestinal endoscopy showed a large, submucosal mass on the gastric antrum. (B) Lesion specimen: tumor texture was medium, dark red fish-like appearance. (C and D) Histological examination of the H&E section from the cell block demonstrated sheets and clusters of spindled to epithelioid cells, numerous neutrophils frequently coexisted with the tumor cells (H&E, ×100, ×400).

**Figure 4.**
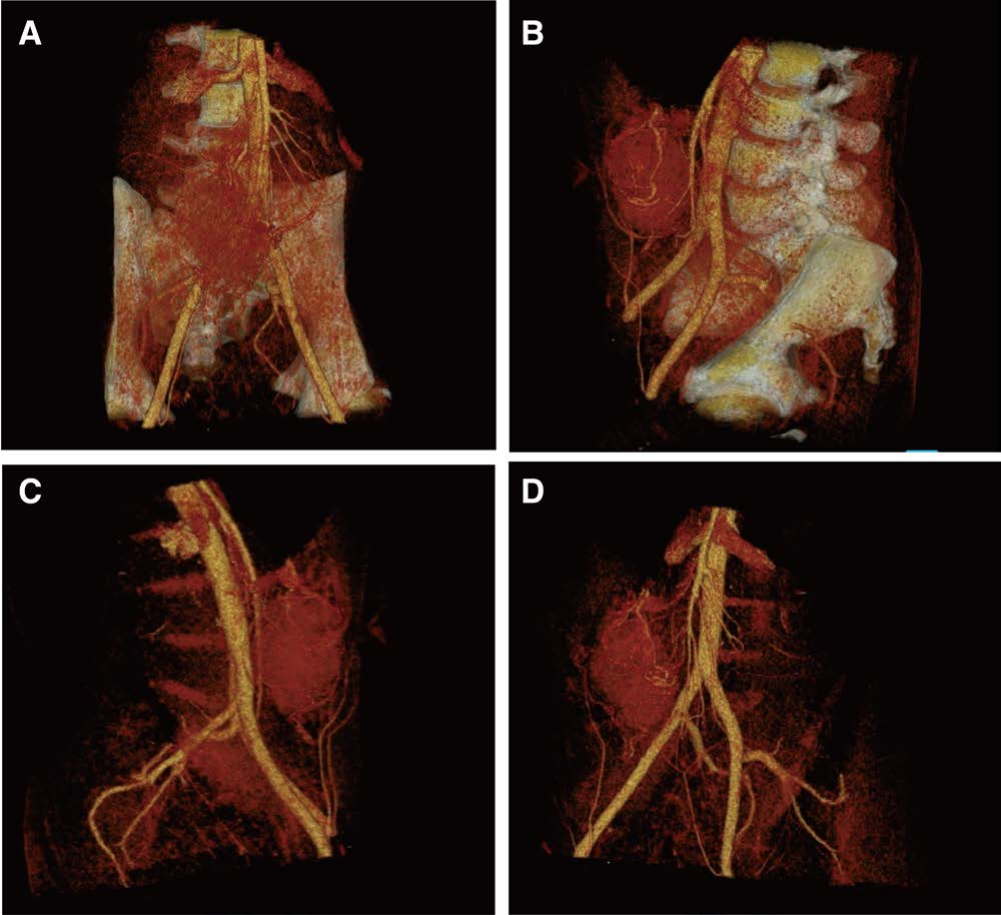
(A–D) Three-dimensional reconstruction images were performed from CT images to demonstrate tumor size and location in anterior and lateral views, respectively. CT = computed tomography.

A contrast-enhanced full abdominal CT scan revealed the presence of a soft tissue mass measuring approximately 68 mm × 47 mm × 91 mm in the right mid-abdomen, with clear boundaries, uneven density within the mass, necrosis and calcifications were not noted, moderate intensified. The lesion was found to be vascularized by the left gastric artery. The boundary between the mass and the gastric antrum was indistinct, and retroperitoneal lymph nodes not swollen (Fig. 1A–C). 18F-FDG positron emission tomography (PET)/CT also showed focal hypermetabolism of the lesion corresponding to the mass in CT, the maximum standardized uptake value (SUVmax) was 25.48 (Fig. 1D–I). Magnetic resonance images (MRI) showed a quasi-circular abnormal signal intensity soft tissue mass in right mid-abdomen. The mass appeared isointense on T1-weighted MRI and heterogeneously slightly hypointense on T2-weighted MRI. On diffusion weighted imaging, the mass was obviously hyperintense. On ADC map, the lesion showed low signal intensity. The mass demonstrated moderate enhancement during the arterial phase of contrast-enhanced imaging, followed by slight uneven enhancement in the portal venous phase and delayed phase (Fig. 2). Upper gastrointestinal endoscopy revealed the presence of a large submucosal mass located on the gastric antrum (Fig. 3A). Three-dimensional reconstruction of the lesion by CT images shows the presence of a soft tissue mass in the right mid-abdomen (Fig. 4).

**Figure 5.**
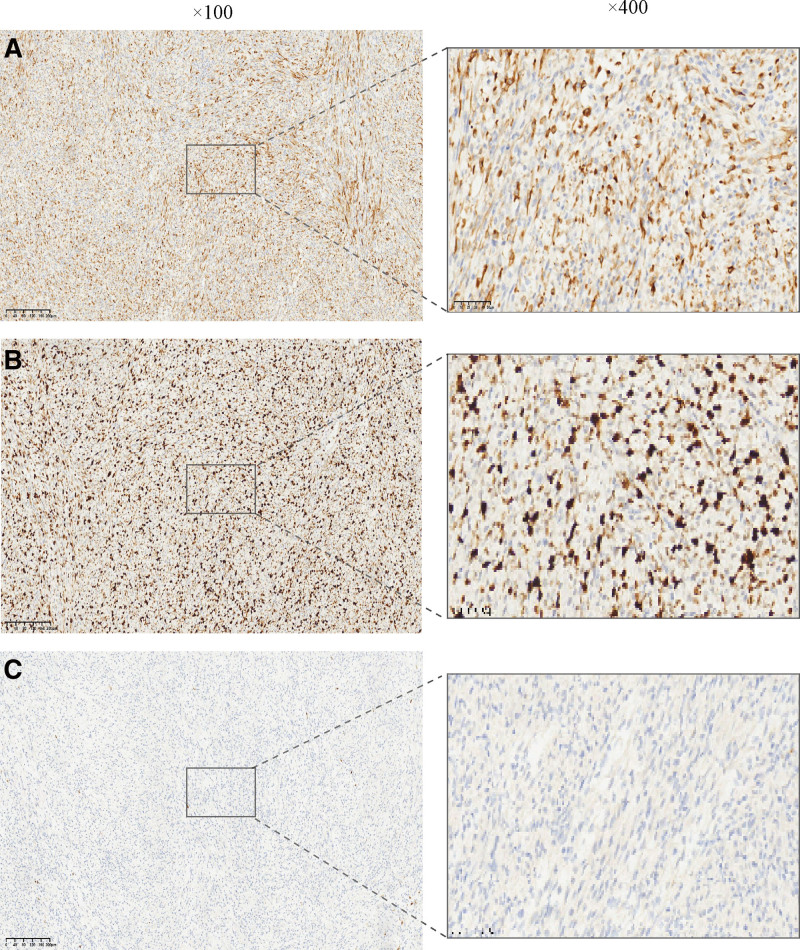
(A) Immunohistochemistry results showed positive CD23. (B) Immunohistochemistry results showed positive Ki67, proliferation index 20%. (C) Immunohistochemistry results showed negative S100 (IHC × 100, ×400). IHC = immunohistochemistry.

The patient presented with clinical symptoms of abdominal pain, leading to a preliminary diagnosis of a gastrointestinal stromal tumor (GIST) based on imaging studies. The patient subsequently underwent radical resection of the gastric tumor and gastrointestinal reconstruction via gastrojejunostomy. Intraoperative findings revealed a well-defined tumor measuring 65 mm × 9 mm, characterized by multinodular growth without necrosis, located in the gastric antrum (Fig. 3B). The tumor was notably surrounded by numerous nutrient vessels. Given its location on the greater curvature of the stomach near the pyloric canal, a distal gastrectomy was deemed appropriate. During the procedure, the lower stomach was transected near the pyloric canal, while the upper stomach was transected approximately 2 cm above the tumor. Gastrointestinal reconstruction was achieved using a Roux-en-Y gastrojejunostomy. No tumor metastasis was found in the perigastric lymph nodes. The presence of patches and clusters of spindle-shaped epithelioid cells and a high number of neutrophils coexisting with tumor cells were revealed by H&E staining of the focal tissue (Fig. 3C and D). Positive for FDCS marker CD23, CD15, CD31, CD3, Cyclin D1, partially positive for CD35, CD68, and were negative for CD21, CK (AE1/AE3), DOG-1, CD34, CD117, SMA, desmin, ALK/P80, SOX10, S-100, CD1a, CD30, CD20 by immunohistochemical staining (Fig. 5A–C). In view of the morphological and immunohistochemical features, a diagnosis of FDCS.

## 3. Discussion

FDCS is a rare neoplasm that arises from the follicular dendritic cells of the germinal centers, primarily located in lymph nodes. Extra-nodal FDCS, particularly in the stomach, is extremely uncommon. Only 7 cases of gastric FDCS have been documented previously., and our case (case 8) adds to this limited body of knowledge (Table 1). The age of patients with gastric FDCS varied from 25 to 64 years, with more male than female patients. The maximum tumor diameter varied from 2 cm to 14 cm in the previous 8 cases, and in our case, the tumor was up to 9 cm in diameter. Clinical presentations included abdominal pain, weakness, anorexia, abdominal fullness, nausea, melena, and palpable mass in previous reports. In one report, the patient experienced mild dyspnea and denied any abdominal pain. In our case, the patient presented with abdominal pain. However, the discussion on the surgical approach and other potential treatment modalities is indeed limited in the literature and in our report. To address this issue, we will explore the full spectrum from diagnosis to treatment.

**Table 1:** Summaries of gastric FDCS cases from the literature.

Case	Reference	Age	Sex	Manifestation	Site	Maximal size (cm)	Treatment
1	Han et al	40	F	Melena, dizziness	Lesser curvature	2.5	Surgery
2	Geerts et al	45	M	NA	Lesser curvature	12	Surgery
3	Shaw et al	60	F	Crampy epigastric pain	Pylorus	2	Surgery
4	Wang et al	53	F	Debilitation, abdominal distention, anepithymia	NA	14	Surgery, radiotherapy, hemotherapy
5	Shacklette et al	31	M	Abdominal pain	Greater curvature	10.6	Surgery
6	Farah et al	36	F	Mild dyspnea	NA	12	Surgery
7	Zhan et al	64	F	Abdominal pain, melena	Lesser curvature	9	Surgery
8	*	25	M	Abdominal pain	Antrum	9	Surgery

F = female, FDCS = follicular dendritic cell sarcoma, M = male, NA = not available.

* Case 8 is our patient.

FDCS in the abdomen is a rare soft-tissue tumor that is often overlooked in imaging evaluations. However, the imaging features of gastric FDCS have only been reported in a limited number of cases due to its rarity. Previous research has primarily focused on clinicopathological features, etiology, and prognosis of FDCS. In 2016, researchers showed CT findings in 5 patients with abdominal FDCS, providing valuable insights into the imaging characteristics of this rare entity.^[9]^ For our case, MRI showed the mass was isointense on T1-weighted MRI and heterogeneously slightly hypointense on T2-weighted MRI. On diffusion weighted imaging, the mass was obviously hyperintense. On ADC map, the lesion showed low signal intensity. Had moderate enhancement in the arterial phase, slight uneven enhancement in the portal venous phase and delayed phase. CT scan showed a soft tissue mass in the right mid-abdomen, with clear boundaries, uneven density within the mass, moderate intensified, but necrosis and calcifications were not noted. No abnormalities were found in the posterior peritoneal lymph nodes. However, in cases involving tumors in the abdomen, heterogeneity was commonly observed due to intratumoral hemorrhage and necrosis, as reported in the literature. The degree of tumor necrosis varied in these cases, and larger tumor sizes were more prone to necrosis.^[10–13]^ Additionally, a portion of the cases demonstrated calcification within the tumor.^[9–13]^ In our case, the volume of the mass was not significant, and the presence of necrosis or calcifications was infrequent.

In discussing the treatment of FDCS, we recognize the need to explore in greater depth the options and efficacy of different therapeutic approaches. FDCS, as a rare tumor, has a relatively limited number of therapeutic strategies. Although FDCS usually presents as slow-growing, about half of the patients will experience local recurrence or even develop an aggressive course with distant metastases to the lungs, liver and lymph nodes. Currently, radical surgical resection is considered the treatment of choice for FDCS, but the choice of surgical approach needs to be based on the location and size of the tumor and the overall condition of the patient. The imaging features described above can pose a challenge in making a preoperative diagnosis of FDCS, as there are several potential differential diagnoses to consider, including GIST and Castleman disease. These diseases often present as large, well-defined masses with areas of necrosis, hemorrhage, or calcifications. In some cases, the intense enhancement of the tumor, similar to that of an adjacent major blood vessel, has been noted, drawing parallels to Castleman disease. Previous studies have also suggested a possible association between FDCS and hyaline vascular Castleman disease.^[13,14]^

In this case, we opted for a distal gastrectomy due to the tumor’s location on the greater curvature of the stomach near the pyloric canal. This surgical approach was chosen to ensure complete removal of the tumor and any potentially involved surrounding tissues, while preserving sufficient gastric tissue to maintain digestive function. During the procedure, we meticulously dissected and managed the trophoblastic vessels surrounding the tumor to minimize intraoperative bleeding and ensure complete resection. We then transected the upper stomach approximately 2 cm above the tumor and the lower stomach near the pyloric canal to establish adequate safety margins. This approach allowed us to achieve radical resection of the tumor while preserving enough gastric tissue to maintain the patient’s digestive and nutrient absorption functions. Postoperatively, gastrointestinal reconstruction was performed using a Roux-en-Y gastrojejunostomy, which helps reduce the reflux of bile and pancreatic fluid into the stomach, thereby minimizing the risk of postoperative complications. Additionally, this anastomosis helps maintain the continuity of the gastrointestinal tract and improves the patient’s quality of life.

The diagnosis of FDCS relies on histopathologic and immunohistochemical examinations. Immunohistochemical markers such as CD21, CD23, and CD35, which are characteristic of dendritic cells, are commonly used. However, in gastrointestinal FDCS, the loss of one or more FDC markers can occur. In this particular case, the tumor cells expressed CD23, CD15, CD31, CD3, CD35, CD68, and cell cycle protein D1. Therefore, the combination of histomorphology and immunohistochemistry is crucial for the diagnosis of FDCS, as these features can vary.

Despite the lack of robust evidence supporting the use of adjuvant therapy for FDCS, radiotherapy may be considered in cases with positive surgical margins or high-risk features, such as large tumor size or deep invasion. Chemotherapy, utilizing agents like anthracyclines and isocyclophosphamide, has shown limited effectiveness due to the relative chemoresistance of FDCS. The potential roles of targeted therapies and immunotherapy in the treatment of FDCS are still under investigation. Consequently, radical resection remains the treatment of choice for FDCS.

In summary, FDCS is a rare and often underrecognized neoplasm, particularly when it arises in extranodal locations. In this case report, we present a unique case of primary FDCS occurring in the stomach of a young male patient. To the best of our knowledge, this is the first report describing the imaging features of FDCS in the stomach using a combination of CT, PET/CT, endoscopy, and MR. These imaging modalities played a crucial role in the diagnosis of FDCS, and their combination can aid in accurate diagnosis and management of this rare tumor.

## Author contributions

**Supervision:** Weihua Gong.

**Writing – original draft:** Xiujuan Sun, Chengyu Hu.

**Writing – review & editing:** Xiujuan Sun, Chengyu Hu.
